# Improved Biocompatibility and Osseointegration of Nanostructured Calcium-Incorporated Titanium Implant Surface Treatment (XPEED^®^)

**DOI:** 10.3390/ma17112707

**Published:** 2024-06-03

**Authors:** Kyung Ran Yang, Min-Ho Hong

**Affiliations:** 1 Daegu Mir Dental Hospital, Jung-gu, Daegu 41934, Republic of Korea; dryangkr815@gmail.com; 2 Department of Dental Laboratory Science, College of Health Sciences, Catholic University of Pusan, 57 Oryundae-ro, Geumjeong-gu, Busan 46252, Republic of Korea

**Keywords:** titanium implant, nanosurfaces, implant surface, histomorphometry, osseointegration

## Abstract

Surface treatment of implants facilitates osseointegration, with nanostructured surfaces exhibiting accelerated peri-implant bone regeneration. This study compared bone-to-implant contact (BIC) in implants with hydroxyapatite (HA), sand-blasted and acid-etched (SLA), and SLA with calcium (Ca)-coated (XPEED^®^) surfaces. Seventy-five disk-shaped grade 4 Ti specimens divided into three groups were prepared, with 16 implants per group tested in New Zealand white rabbits. Surface characterization was performed using X-ray diffraction (XRD), field emission scanning electron microscopy (FE-SEM), digital microscopy, and a contact angle analyzer. Cell viability, proliferation, and adhesion were assessed using MC3T3-E1 cells. Apatite formation was evaluated using modified simulated body fluid (m-SBF) incubation. After 4 weeks of healing, the outcomes reviewed were BIC, bone area (BA), removal torque tests, and histomorphometric evaluation. A microstructure analysis revealed irregular pores across all groups, with the XPEED group exhibiting a nanostructured Ca-coated surface. Surface characterization showed a crystalline CaTiO_3_ layer on XPEED surfaces, with evenly distributed Ca penetrating the implants. All surfaces provided excellent environments for cell growth. The XPEED and SLA groups showed significantly higher cell density and viability with superior osseointegration than HA (*p* < 0.05); XPEED exhibited the highest absorbance values. Thus, XPEED surface treatment improved implant performance, biocompatibility, stability, and osseointegration.

## 1. Introduction

Titanium (Ti) and its alloys are widely used as implant materials due to superior biocompatibility and better osseointegration than other metals [1]. They are promising candidates for orthopedic applications (artificial joints) and dentistry (fixture materials for dental implants) [2,3]. Dental implant materials should be non-toxic, non-allergenic, and corrosion-resistant and exhibit physicochemical stability following oral exposure [2]. In particular, commercially pure Ti (cp Ti) is the most widely used material for dental implants due to its excellent mechanical properties (tensile strength up to 850 MPa and elongation of ~10%) and biocompatibility [3].

First-generation dental implants were cylindrical with a mechanically machined surface [4] fabricated using CNC (Computer Numerical Control) machining (or subtractive machining technology). Since then, clinicians and researchers have been striving to achieve better osseointegration, which refers to the functional bone formation that occurs in direct contact with the implant [2]. Successful osseointegration after implantation is crucial in determining long-term implant survival, and a stable bond between the implant and surrounding tissue plays a significant role [5]. Therefore, implant surface characteristics should enhance cell adhesion and the interaction between implant surfaces and surrounding bone. The characteristics are determined by various factors, such as biocompatibility and surface roughness of implant materials [6,7,8,9], with surface roughness levels being a critical factor in biomechanics. Surface roughness can be classified into macroscale, microscale, and nanoscale surface types [10]. Studies investigating initial fixation and mechanical aspects of implant stability with different surfaces have shown that surfaces with moderate roughness perform better than smooth surfaces [11]. Implant surfaces with a nanoscale size exhibit superior protein adsorption and enhance osteoblast attachment and proliferation, contributing to early osseointegration [11]. Implant surface roughness ranges from 1 to 23 μm, and measurement methods usually have an accuracy of less than 1 μm [12,13,14,15].

Implant geometry and surface topography are critical for short-term and long-term success. Recent research has paid significant attention to developing implant surfaces promoting faster and improved osseointegration [5,16,17]. Various surface modifications, including hydroxyapatite (HA) coating, sandblasted large-grit acid-etching (SLA), chemical vapor deposition (CVD), plasma spraying, plasma electrolytic oxidation (PEO), electrical discharge machining (EDM), and their combinations, have been developed [18,19]. Ti surfaces are coated with HA to improve the surface reactivity of their biologically inert surfaces, using the popular plasma spray HA-coating technology. According to Barrere et al., the HA-coated implant surface improves osseointegration compared to the noncoated group [20]. However, other studies have reported that the survival rates of implants fabricated by the HA-coating treatment method are similar or lower than those for conventional implants [21]. Moreover, the plasma-based HA coating method also presents certain disadvantages. The coated HA layer can delaminate from the Ti surface as it adheres to the bone tissue [22].

One of the most commonly used commercial methods to enhance the surface roughness of Ti implants is the SLA process, which involves blasting the Ti alloy surface with coarse aluminum oxide (Al_2_O_3_) abrasive particles (0.2–0.5 mm), followed by etching using strong acids [23]. The SLA technique increases the microroughness and hydrophilicity of the Ti surface. The implant is soaked in an etching solution composed of sulfuric acid (H_2_SO_4_) and hydrochloric acid (HCl) to create microscale and nanoscale surface irregularities [21]. This improved surface maintains a wide surface area and high surface energy during the bone remodeling phase of osseointegration, promoting protein adsorption, cell adhesion, and mechanical bonding with surrounding bone [11,23]. However, SLA surface treatment may leave residual strong acids on implant surfaces [24], posing a safety hazard. Thus, the “optimal” surface treatment method remains elusive, and the rate of early osseointegration is a critical criterion [25]. Research on surface treatment of Ti implants has attempted to enhance the biological response between tissues and implant surfaces and maximize new bone formation at the bone–implant interface after implantation [26]. The surface modification of Ti implants can enhance the biological response of implant surfaces and surrounding tissues, leading to increased osseointegration and improved clinical outcomes. Recently, researchers introduced nanostructured, calcium (Ca)-enriched surface (XPEED^®^) Ti implants, enhancing BIC and reducing initial healing time [27]. This surface modification exhibits higher biocompatibility than conventional Ti implant surface modifications because it eliminates the risk of acidic residues on the Ti alloy surface and provides Ca ions to the surrounding tissue [27]. Animal studies show that XPEED significantly improves BIC [28].

In vitro studies have shown that surface modification by Ca ions increases the growth of osteoblast-like cells and promotes HA deposition on Ti surfaces in simulated body fluid [29]. Moreover, the cell adhesion effect on the Ca-adsorbed Ti surface decreases in human gingival fibroblasts and MG-63 cells but increases in human osteoblast-like cells [29]. Several in vivo studies have reported that adding Ca to Ti implants via thermal treatment stimulates osseointegration, resulting in a higher BIC percentage (BIC%) than untreated Ti implants in a rabbit model [30,31]. After adding Ca via thermal modification, surface roughness is modified only at the nanoscale level and not at the micron level [30,32].

Clinical studies have objectively evaluated osseointegration. However, further research is needed to ensure the reliability of the osseointegration of Ca-coated SLA surfaces (XPEED^®^). The BIC technique is widely accepted as an objective method to evaluate the performance of dental implant surfaces [32,33,34,35,36]. It allows for the monitoring, visualization, and assessment of bone growth on implant surfaces and has been used in numerous studies [37,38]. This study compared the BIC of implants with HA, SLA, and SLA with a Ca-coated (XPEED^®^) surface. The null hypothesis was that no statistically significant differences are present in the BIC ratio for the HA-blasting surface, SLA surface, and SLA with Ca-coated (XPEED^®^) surface at 4 weeks of healing.

## 2. Materials and Methods

### 2.1. Surface Treatment of Samples

Seventy-five disk-shaped (diameter: 10 mm; thickness: 3 mm) grade 4 Ti specimens (MEGAGEN Implant Co., Ltd., Daegu, Republic of Korea) were prepared and divided into three groups based on processing: HA, SLA, and SLA with Ca-incorporated (XPEED^®^) surfaces (Table 1).

The HA group specimens were used to improve affinity to bone, including sandblasting to enhance the surface roughness using HA (Ca_10_(PO_4_)_6_(OH)_2_). The SLA group specimens were prepared by blasting the surfaces using 100–150 μm of aluminum oxide (Al_2_O_3_) to form large craters and an etching solution mixed with HCl (CAS No. 7647-01-0, Duksan Pure Chemicals Co., Ltd., Ansan-si, Gyeonggi-do, Republic of Korea) and H_2_SO_4_ (CAS No. 7664-93-3, Duksan Pure Chemicals Co., Ltd., Ansan-si, Gyeonggi-do, Republic of Korea) to produce micropits. For XPEED groups, the surfaces were blasted using aluminum oxide and treated with an etching solution mixed with HCl/H_2_SO_4_/H_2_O at 100 °C for 10 min, followed by washing and drying in an automatic vacuum ultrasonic cleaner. Next, after dissolving 0.2 M NaOH in triple-distilled water, adding 2 mM CaO, and stirring for 1 h in an argon atmosphere, the dried samples were placed in this solution in a hydrothermal reactor and left to react at 200 °C for 2 h, followed by washing in an automatic vacuum ultrasonic cleaner. Furthermore, 16 implant samples were prepared per group for animal testing (Table 1). The implant samples were divided into three groups and prepared by the same surface treatment as the disk specimen preparation process. All samples were used for testing after gamma irradiation.

### 2.2. Surface Characterization

The three groups were analyzed using X-ray diffraction (XRD) (MAXIMA-X XRD-7000, Shimadzu Corp., Kyoto, Japan) with Cu Kα radiation (λ = 0.1541 nm). The scan range was 20–80 degrees at a 2θ angle, and the voltage was set at 30 kV. The surface topography of the Ti samples was examined using field emission scanning electron microscopy (FE-SEM) (SU8010, Hitachi, Tokyo, Japan). An acceleration voltage of 15 kV in the secondary electron mode at high vacuum was used for the analysis. The samples were imaged at magnifications of 5000×, 10,000×, and 50,000×.

The surface roughness of the samples was measured using a digital microscope (VHX-7000, Keyence, Itasca, IL, USA) with a 300× magnification. A 10 mm diameter disc was placed in the center of a 170 mm diameter circular plate to measure roughness. The disc-shaped specimen was placed in the center, with a distance of 80 mm in both directions and a tilt angle of 0 degrees. The measurement was taken in the vertical direction using autofocus, with no measurement taken in the lateral direction. After the measurement, the image resolution was 2880 × 2160 pixels. 3D profile measurement module (VHX-H5M, Keyence, Itasca, IL, USA) software was used to analyze the surface roughness data, and 3D images were captured for each sample. Twenty images of size 630 μm × 630 μm were obtained for each sample and aligned to obtain surface roughness data and 3D images (n = 6). Ten random spots were observed on each of the three samples from each group, and the average Ra (mean surface profile roughness) and surface texture scan Sa (the center plane average) values were calculated.

The wetting properties of the three different surfaces were evaluated using the sessile drop method with a contact angle analyzer (Phoenix-MT(A), SEO Co., Ltd., Suwon-si, Gyeonggi-do, Republic of Korea). At room temperature, droplets of equal volume (1.0 μL) were dispensed onto the specimen, and the left and right contact angles were measured (n = 6). In addition, a preliminary test was conducted to ensure the purity of distilled water and syringe cleanness. The contact angle was analyzed using an image analysis program (Surfaceware 9; SEO Co., Ltd., Suwon-si, Gyeonggi-do, Republic of Korea) [18]. The surface chemistry was characterized using X-ray photoelectron spectroscopy (XPS, Nexsa, Thermo Fisher, Oxford, UK) with Al Kα radiation. After acquiring XPS data, samples were cleaned with argon sputtering for 30 s to remove surface contaminants and measure chemical composition, binding energies, and peak areas. The XPS analysis was used to determine the elements present in the surface layers. The surfaces were removed by argon sputtering, and the separated interface layers were analyzed using the XPS depth profile technique to quantify the elemental distribution throughout each surface.

### 2.3. Cell Viability

#### 2.3.1. Cell Cytotoxicity

The culture medium used for cultivating MC3T3-E1 cells (CRL-2593, ATCC, Manassas, VA, USA), a mouse calvaria-derived osteoblast-like cell line, consisted of alpha minimum essential media (α-MEM, Gibco, Grand Island, NY, USA) supplemented with 10% fetal bovine serum (FBS, Gibco, Grand Island, NY, USA) and 1% penicillin G–streptomycin (WelGENE, Gyeongsan-si, Gyeongsangbuk-do, Republic of Korea). Cell suspensions were seeded into a 24-well plate at a concentration of 5 × 10^4^ cells/well and cultured at 37 °C in a 5% CO_2_ incubator while simultaneously conducting elution from Ti disks at 37 °C. After 24 h, the eluate was replaced, and culturing was continued for another 24 h. An MTT (3-(4,5-dimethylthiazol-2-yl)-2,5-diphenyltetrazolium bromide) reagent was added to each well to induce formazan formation, followed by extraction with dimethyl sulfoxide (DMSO, Sigma Aldrich, St. Louis, MO, USA), and absorbance was measured at 570 nm using an enzyme-linked immunosorbent assay (ELISA, analyzer Sunrise, Tecan Trading AG, Männedorf, Switzerland) reader. Cell viability above 70% indicated biocompatibility [39].

#### 2.3.2. Cell Proliferation

MC3TC-E1 cells (CRL-2593, ATCC, Manassas, VA, USA) were used for cell proliferation experiments, and MTT was used as a staining reagent. Cell proliferation tests were conducted using methods similar to those used in cytotoxicity. Cells were cultured for 1, 3, and 5 d. The cells attached to the disc were rinsed in a serum-free medium to remove poorly attached cells. The MTT solution was administered to each disk to create an environment for formazan formation, and the absorbance was measured at 570 nm using a spectrophotometer [11,28].

#### 2.3.3. Cell Adhesion

Cells were cultured in a 24-well plate at a concentration of 5 × 10^4^ cells/well for 24 h. Cells attached to the disk were fixed with 4% formaldehyde for 30 min and dehydrated gradually with increasing ethanol concentrations. After drying for a certain period, platinum coating was applied, and the attachment morphology of cells was examined using SEM [11].

### 2.4. Apatite-Forming Ability Assessment

Modified–simulated body fluid (m-SBF, Sigma-Aldrich, St. Louis, MO, USA) was prepared as follows, referring to the description by Oyane et al. [40]. First, the corresponding reagents were dissolved in distilled water in the order shown in Table 2. The pH was adjusted to 7.4 using 2-(4-(2-hydroxyethyl)-1-piperazinyl) ethanesulfonic acid (HEPES, Sigma-Aldrich, St. Louis, MO, USA) and NaOH (CAS No. 1310-73-2, Duksan Pure Chemicals Co., Ltd., Ansan-si, Gyeonggi-do, Republic of Korea). The solution was replaced twice a week. After 7, 10, 12, and 14 d, the implant was washed with distilled water and dried for 24 h in an oven. The morphology of apatite was analyzed using SEM equipped with an energy-dispersive X-ray spectrometer (SEM/EDS, S-4800, Hitachi, Tokyo, Japan).

### 2.5. Animal Tests and Surgical Procedures [31,41]

This study used 12 3.5-kg New Zealand male white rabbits. The experiment received approval from the Animal Experiment Ethics Committee of Kyungpook National University Hospital. We followed domestic regulations on the care and use of experimental animals (equivalent to the NIH guidelines) (NIH Publication No. 85-23, revised in 1985). General anesthesia was induced by mixing 1.3 mL of ketamine (100 mg/mL, Ketara, Yuhan Corporation, Seoul, Republic of Korea) and 0.2 mL of xylazine (7 mg/kg of body weight; Rompun, Bayer Korea, Seoul, Republic of Korea). The surgical procedure was performed on the medial aspect of the proximal tibia. Before draping, the area was shaved and cleansed with a solution of iodine and 70% ethanol. After achieving hemostasis, local anesthesia was administered using an additional 1 mL of 2% lidocaine (1:100,000 epinephrine; Yuhan, Republic of Korea). The surgical area was prepared using aseptic techniques, and an incision was made through the skin, fascia, and periosteum of the medial aspect of the proximal tibia to expose the region. The osteotomy for implant placement was performed with a 3.6 mm diameter drill using conventional methods. Dental implant drilling was performed after sterilizing the drill with the saline solution. A set of HA, SLA, and XPEED implants was randomly placed in the right and left legs. All implants were placed in the tibia. The implants of HA (n = 16), SLA (n = 16), and XPEED (n = 16) groups were inserted using the recommended torque (35 N cm). All implants penetrated only the first cortical bone. After surgery, the incisions were sutured with Vicryl (Ethicon, Somerville, NJ, USA). To prevent infection and control pain post-surgery, antibiotics (Baytril, Bayer Korea) and analgesics (Nobin, Bayer Korea) were injected into the muscles for 3 d. At 4 weeks post-surgery, the animals were euthanized by an intravenous injection of air under general anesthesia.

### 2.6. Removal Torque Tests [41,42]

Removal torque tests were conducted to evaluate implant stability. The measurements are expressed in Newton centimeters (N cm) to reflect interfacial shear strength. The results obtained from the removal torque tests indicated the integration strength of the implants within the bone tissue. The tibiae containing the implants were removed as blocks and firmly secured in a vise for removal torque measurements. The maximum removal torque force was measured using a digital torque meter (MG series, Mark-10 Corporation, New York, NY, USA). All measured maximum torque values when starting rotation in the opposite direction were recorded. This study evaluated HA, SLA, and XPEED implants. At approximately 4 weeks after implantation, the removal torque values between samples placed in the tibia and fibula were measured and compared.

### 2.7. Histomorphometric Evaluation: BIC and Bone Area (BA) Tests [31,41]

Fixed samples were the basis for measuring BIC, and BA was evaluated by analyzing the remaining bone on the implant surface after the removal torque analysis. Samples of the implants and surrounding tissues were retrieved from the tibia for histological assessment, fixed in 10% neutral buffered formalin for 24 h, dehydrated in a graded series of alcohols, and embedded in methyl methacrylate resin. Furthermore, 20 µm thick sections were prepared using a grinding machine (Exakt 310 CP series, Exakt Apparatebau, Norderstedt, Germany), with the center of the implant included in each section. All sections were stained with Villanueva Osteochrome Bone Stain (Polysciences Inc., Warrington, PA, USA) before microscopic examination. BIC% and BA percentage (BA%) values were measured over all threads. BIC% was the percentage of the length of mineralized bone contacting the implant surface directly, and BA% was evaluated by measuring the amount of mineralized bone inside all threads. BIC% and BA% were measured using an image analysis program (Analysis TS Auto; Olympus).

### 2.8. SPSS One-Way Analysis Test

The results for surface roughness, contact angle values, cell viability, removal torque values, and histomorphometric data tests were examined for normal distribution using the Shapiro–Wilk test and equal variances using the Levene test and then analyzed using 1-way ANOVA and the post hoc Tukey multiple comparison test (α = 0.05). Statistical analyses were performed using SPSS Statistics v17.0; SPSS Inc. (Chicago, IL, USA).

## 3. Results and Discussion

**Figure 1:**
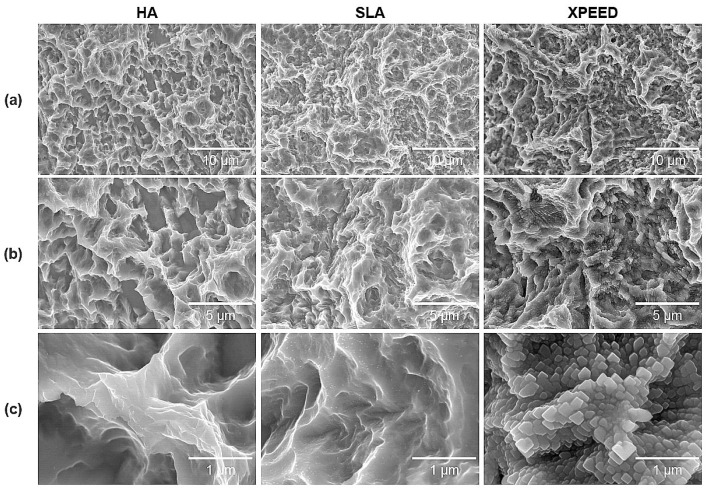
SEM images of Ti alloys: (**a**) 5000× magnification, (**b**) 10,000× magnification, and (**c**) 50,000× magnification. Scale bars are (**a**) 10 μm, (**b**) 5 μm, and (**c**) 1 μm. HA: hydroxyapatite sandblasted; SLA: alumina sandblasted + acid-etched; XPEED: alumina sandblasted + acid-etched + Ca-coated.

**Figure 2:**
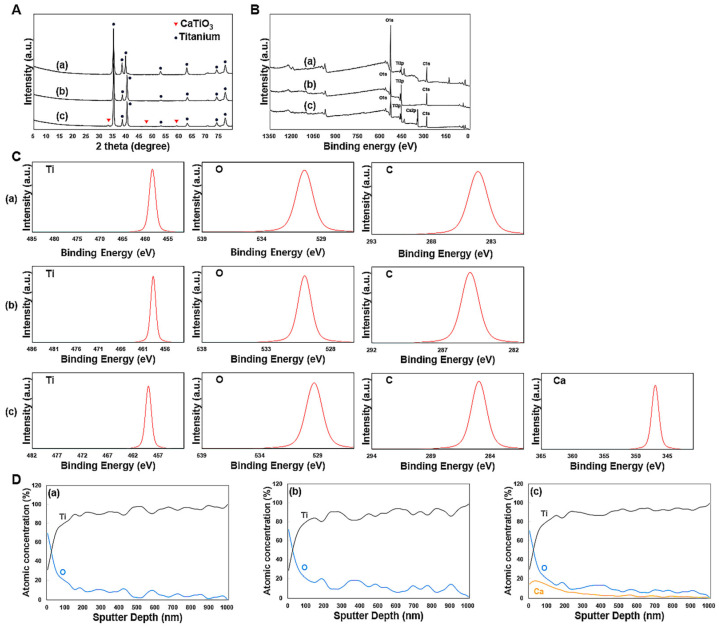
X-ray diffraction patterns (**A**), XPS survey spectra (**B**), deconvolution spectra (**C**), and Auger electron spectroscopy depth profile (**D**) of Ti surfaces: (**a**) HA, (**b**) SLA, and (**c**) XPEED. HA: hydroxyapatite sandblasted; SLA: alumina sandblasted + acid-etched; XPEED: alumina sandblasted + acid-etched + Ca-coated.

Figure 1 shows the microstructures of each group for the three surface treatments. SEM images at low magnification (5000× and 10,000×) showed that all groups exhibited microtopography of porous structures with irregular pores and sharp edges. Additionally, there were no morphological differences at the micron scale. However, in the high magnification (50,000×) SEM image, the XPEED group, which received hydrothermal treatment in the Ca-coated treatment step, demonstrated a nanostructured Ca-coated surface (Figure 2). These nanoscale structures may play a role in inducing early bone cell formation by promoting protein interactions [43].

Figure 2 presents the XRD and XPS test results for the Ti alloys. The XRD pattern revealed similarities between the HA and SLA groups (Figure 2A). In contrast, the XPEED group showed a crystalline CaTiO_3_ (JCPDS No. 22-0153) layer on the surfaces of hydrothermally treated Ti alloys (Figure 2A). The binding energy ranges for the Ti2p, O1s, and C1s elements in each sample were within the characteristic ranges of 457–465 eV for Ti2p, 529–534 eV for O1s, and 284–288 eV for C1s (Figure 2B,C). Specifically, the O1s peak for the XPEED sample was somewhat broadened compared to the HA and SLA samples, and a new sub-peak around 531 eV was generated, probably due to the generation of (OH)s and C–O bonds (Figure 2B,C) [28]. A Ca2p peak was generated within the binding energy range of 342–352 eV due to the effect of hydrothermal treatment with the CaO mixture (Figure 2B,C). The XPS analysis was used to determine the elements in the surface layers. The surfaces were removed by argon sputtering, and the separated interface layers were then analyzed using the XPS depth profile technique to quantify the elemental distribution throughout each surface (Figure 2D). The results confirmed that the graph shapes of Ti2p and O1s were very similar on the HA and SLA surfaces. These results could be attributed to the surfaces of the HA and SLA samples that were etched at a similar rate while undergoing strong acid etching during the process. In particular, the Ca element (absent from the HA and SLA surfaces) showed even distribution on the XPEED Ti surface. All surfaces showed an increased proportion of oxygen on the outermost surface at 0 nm and a gradual decrease to almost nonexistent levels below 100 nm, indicating that the thin TiO_2_ layer decreased significantly when going deeper into the implants. The presence of Ca on the XPEED surface also continued in trace amounts below 100 nm and was detected up to 600 nm, confirming that Ca was coated on the implant exterior and incorporated deep inside.

**Figure 3:**
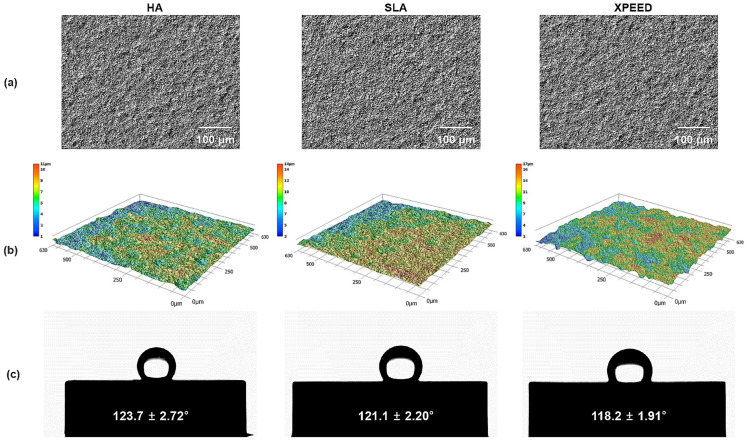
Optical images (**a**), 3D topography (**b**), and contact angle (**c**) images of HA, SLA, and XPEED groups; (**a**) 300× magnification; scale bar = 100 μm. HA: hydroxyapatite sandblasted; SLA: alumina sandblasted + acid-etched; XPEED: alumina sandblasted + acid-etched + Ca-coated.

Table 3 summarizes the results of the surface roughness and contact angle values for the three Ti alloys. Figure 3 also displays representative images of the surface roughness and contact angle. The surface roughness and contact angle showed similar values in the HA, SLA, and XPEED groups. According to previous studies [11,44], the bonding force between the implant and the bone increases with surface roughness in the 1–10 μm range. Moreover, nanometer roughness in the 1–100 nm range improves osteoblast adhesion and proliferation and plays an important role in protein adsorption. A moderately rough surface (Ra and Sa: 1.0–2.0 µm) was created on implant surfaces in all groups (Figure 1 and Figure 3). The contact angle measurement results showed no statistically significant differences among the three groups (*p* > 0.05). In contrast to previous studies [45], all groups exhibited high contact angles, suggesting that the samples used in this study experienced reduced hydrophilicity due to gamma irradiation, leading to increased contact angles in all groups [46].

**Figure 4:**
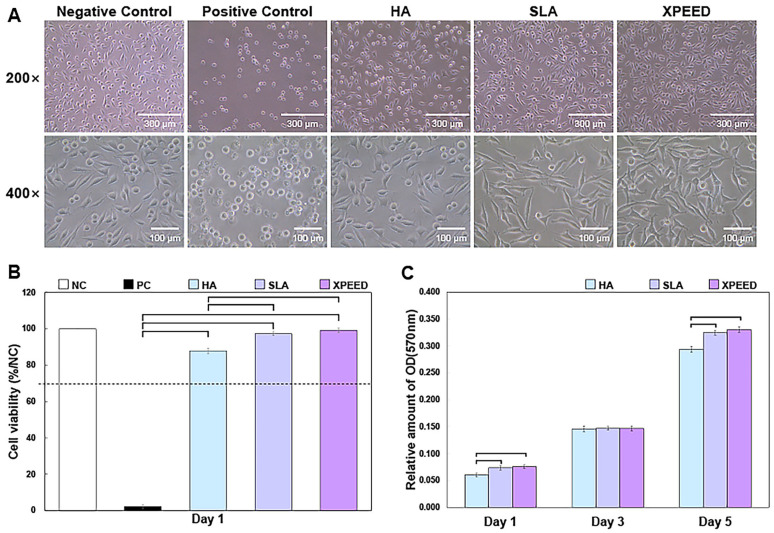
Phase-contrast microscopic images of MC3T3-E1 cells after 1 d of culture (**A**), cell viability results after 1 d of culture (**B**), and cell proliferation—the absorbance OD values for 1, 3, and 5 d in vitro (**C**). NC, negative control; PC, positive control; HA, hydroxyapatite sandblasted; SLA, alumina sandblasted + acid-etched; XPEED, alumina sandblasted + acid-etched + Ca-coated. The analysis of results was based on the ISO 10993-5:2009 standard guidelines, in which a biomaterial is considered cytotoxic for cell viability below 70%. The dashed lines represent the ISO 10993-5 cut-off level (70%). In the bar graph, significant differences are denoted with horizontal lines connecting the bars (*p* < 0.05).

Figure 4 shows the phase-contrast microscopy images of MC3T3-E1 cells (CRL-2593, ATCC, Manassas, VA, USA) after 1 d of culture and cell viability results after 1 d of culture and cell proliferation—the absorbance OD values for 1, 3, and 5 d. On the plate from which each sample was eluted, cells also showed various shapes depending on the sample. HA showed many relatively round-shaped cells. When confirmed at 400× magnification, the ratio of round cells to expanded cells appeared to be divided by 50%. In the SLA sample, many cells grew slightly longer than the HA. Approximately 89% of the cells were elongated, forming filopodia within the reference area. Lastly, in XPEED, almost all cells exhibited oval- or spindle-shaped cells. The quantitative value was approximately 90% within the standard area. Initially, the round-shaped cells were almost nonexistent, and the cell body grew into a long oval shape with filopodia expanding in all directions. According to these shape changes, XPEED preserves a more favorable environment for cell growth than HA and SLA.

The XPEED and SLA groups exhibited significantly greater cell density and cell viability (close to the negative control) than the HA groups (*p* < 0.05). There was no significant difference in the cell viability between the XPEED and SLA groups (*p* > 0.05). In ISO 10993-5:2009 [47], a reduction in cell viability exceeding 30% in comparison to non-toxic controls is considered cytotoxic [39]. The absorbance analysis was performed for a quantitative evaluation of the cell growth rate. This analysis method involved treating cells with an eluate and checking how well the cells survived in the eluate. The blank test solution was the control, and absorbance values closer to 100% correlated with higher growth rates. According to the experimental results below, the absorbance values for each sample were somewhat different. First, XPEED showed the highest absorbance value at 99.2%, followed by SLA and HA at 97.35% and 87.90%, respectively. These results indicated that cells grew best in XPEED, which provided a favorable environment for cell growth in the following order: SLA and HA. All three conditions provided excellent environments for cell growth, but XPEED exhibited the best cell viability due to the adsorption of Ca ions on the surface. When high concentrations of divalent cations (such as Ca) bind to the surface, they can stimulate integrin-mediated cellular responses, leading to high growth performance in osteoblasts [41,48]. Therefore, the XPEED surface had the highest absorbance because it significantly influenced cell growth due to Ca [29]. The cell proliferation results shown in Figure 4C reveal a 3-fold increase in cell density from d 1 to d 3 on all surfaces. The value reached approximately 0.32 by d 5, which is a 6-fold increase compared to the initial value. Higher cell proliferation was found in the XPEED and SLA groups than in the HA group only on d 1 and 5 (*p* < 0.05). However, there were no statistically significant differences between the groups on d 3 (*p* > 0.05).

**Figure 5:**
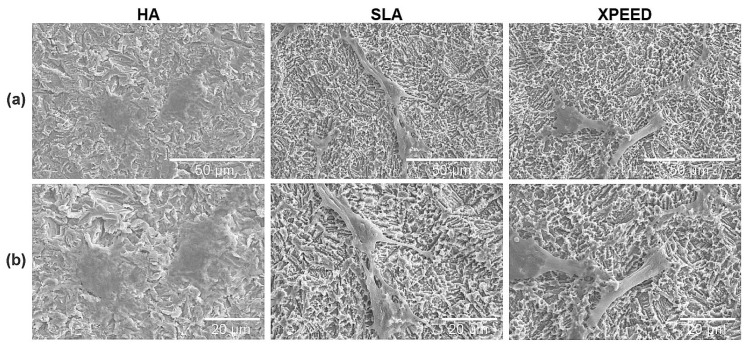
SEM images of MC3TC-E1 cells after the growth of cytoplasm on each surface: (**a**) 1000× magnification and (**b**) 1500× magnification. Scale bars are (**a**) 50 μm and (**b**) 20 μm. HA: hydroxyapatite sandblasted; SLA: alumina sandblasted + acid-etched; XPEED: alumina sandblasted + acid-etched + Ca-coated.

Figure 5 shows SEM images of the cytoplasm growing on the surfaces. The surfaces were observed using SEM, and the morphologies of the attached cells varied according to the surface morphology, with the SLA and XPEED surfaces showing the highest spread of cytoplasm and long, stretched filopodia. According to the literature, nanoscale particle sizes and density affect cell behavior, significantly improve cell and tissue responses, and produce faster osteoblast differentiation, depending on the process adopted [49,50]. Moreover, cell spreading has a crucial effect on cell functions, such as cell proliferation and differentiation. Nanostructures can stimulate osteoblast cells to foster a structure that facilitates cell spreading [51,52]. Thus, the current results showed that the cells grew best on the XPEED surface, which has distinct nanostructures.

**Figure 6:**
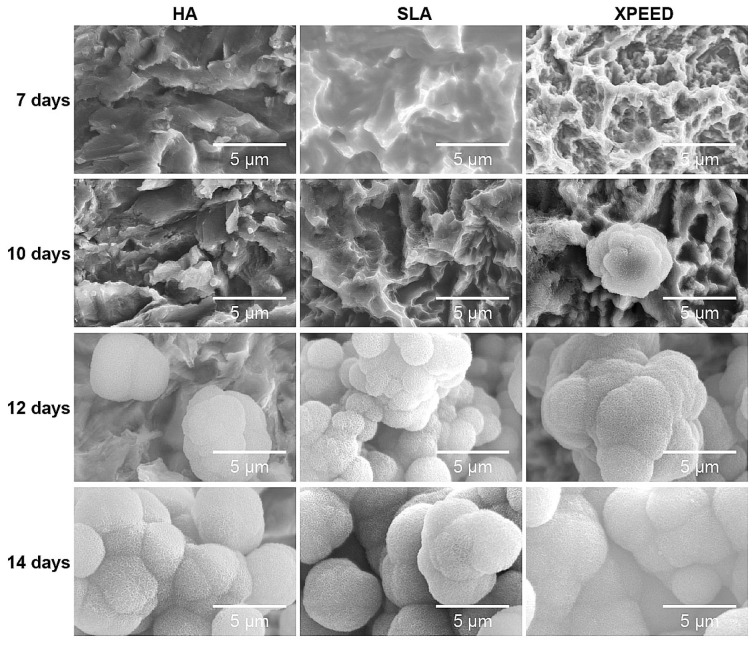
SEM images of the HA, SLA, and XPEED groups incubated in m-SBF for 14 d. The XPEED group showed more and faster apatite formation on the alloy surface than the HA and SLA groups. HA: hydroxyapatite sandblasted; SLA: alumina sandblasted + acid-etched; XPEED: alumina sandblasted + acid-etched + Ca-coated.

To confirm bioactivity and biocompatibility according to surface treatment, apatite formation was confirmed by incubating the samples in the m-SBF solution at 37 °C for 14 d (Figure 6). On the 7th d, apatite formation was not clearly visible on all surfaces, but on the 10th d, apatite formation was clearly visible in XPEED. On the 12th d, the HA, SLA, and XPEED surfaces exhibited the formation of aggregated, 5 μm or larger, spherical apatite particles. When sedimented for 14 d, the entire surface was completely covered with apatite, confirming significant apatite growth. Apatite is composed of Ca^2+^, PO_4_^3−^, and -OH functional groups [53]. The mechanism of apatite formation involves attracting Ca^2+^ ions of m-SBF with –OH and PO_4_^3−^ present on the apatite surface [54]. In the case of XPEED, since Ca^2+^ ions were already held on the surface, Ca^2+^ formed along with m-SBF, suggesting that increased ion concentration resulted in quicker apatite formation than on HA and SLA surfaces.

**Figure 7:**
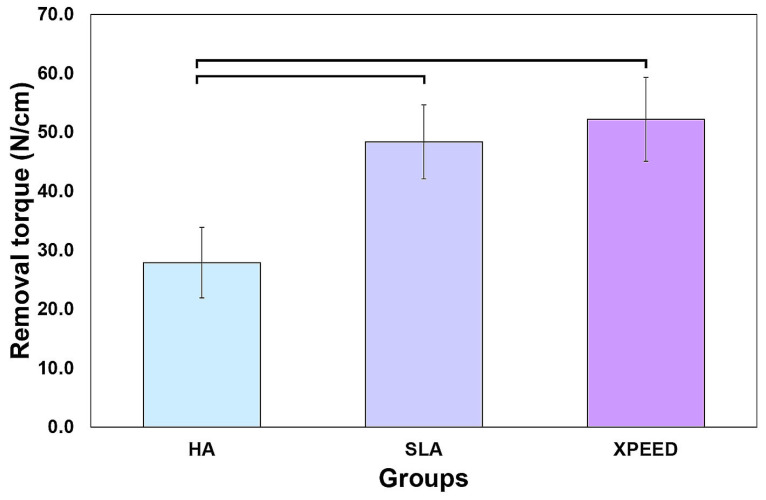
Removal torque values of the HA, SLA, and XPEED implants after 4 weeks of healing. In the bar graph, significant differences are denoted with horizontal lines connecting the bars (*p* < 0.05). HA: hydroxyapatite sandblasted; SLA: alumina sandblasted + acid-etched; XPEED: alumina sandblasted + acid-etched + Ca-coated.

Figure 7 shows the results of measuring the removal torque values approximately 4 weeks after implant placement. XPEED and SLA groups exhibited significantly higher removal torque values than the HA group (*p* < 0.05), with no significant difference in the removal torque values between the XPEED and SLA groups (*p* > 0.05). Factors affecting the removal torque include geometric characteristics, interfacial tissue structure, and the quantity and quality of the surrounding bone. The microscopic morphology of the surface also has a significant influence [28,29,30,31,41]. According to the results of this experiment, a high average surface roughness of XPEED, provided by Ca ions and nanostructures, stimulated cells and improved cell adhesion, which, in turn, affected the bone bonding strength, leading to the highest removal torque values [30,31].

**Figure 8:**
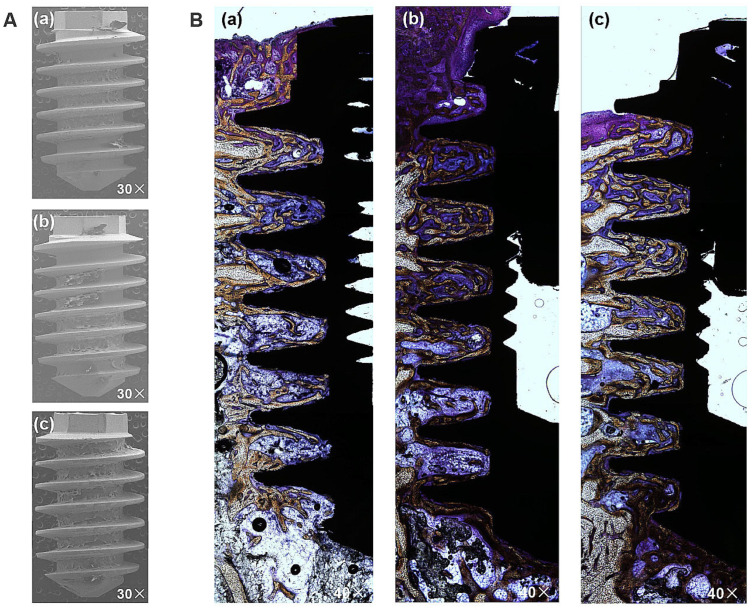
Histological sectional images showing bone contact along Ti implant surfaces after removal at 4 weeks post-placement. A high degree of direct bone contact is seen on the surfaces of the three groups. (**A**) An image of the removed implant; 30× magnification. (**B**) Representative Villanueva Osteochrome Bone Stain images of three groups; 40× magnification. (**a**) HA, (**b**) SLA, and (**c**) XPEED. HA: hydroxyapatite sandblasted; SLA: alumina sandblasted + acid-etched; XPEED: alumina sandblasted + acid-etched + Ca-coated.

The BIC was measured based on fixed sample slides, while the BA was evaluated by analyzing the implant surfaces after removal torquing to observe the amount of remaining bone. The representative images in Figure 8A confirm the attachment of biomaterial to the implant following the removal of the HA, SLA, and XPEED implants in rabbits. Notably, the XPEED implant exhibited more bone and tissue attachment than other groups. All implants showed no signs of inflammation at the BIC, and in particular, the XPEED implant showed continued bone deposition on the surfaces of areas with trabecular loss (Figure 8B). The XPEED implant showed higher BIC in the medullary canal (which was devoid of surrounding bone) than the HA and SLA implants. The XPEED implant surface was more homogeneous and densely mineralized than the HA and SLA implant surfaces. Thus, the XPEED implant surface demonstrated better osteoconductive properties than the HA and SLA implant surfaces.

**Figure 9:**
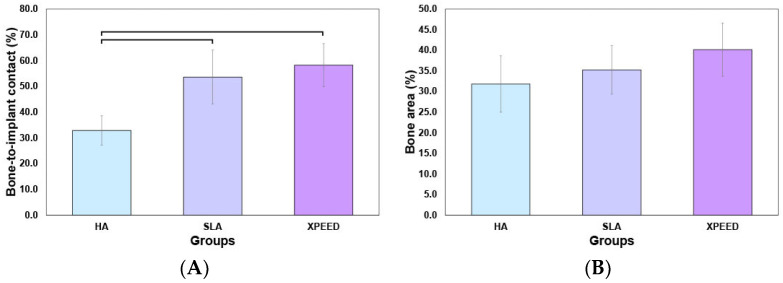
Mean percentages of the bone-to-implant contact (**A**) and bone area (**B**) in all threads of implants 4 weeks after implantation. HA: hydroxyapatite sandblasted; SLA: alumina sandblasted + acid-etched; XPEED: alumina sandblasted + acid-etched + Ca-coated.

Figure 9 displays the BIC and BA results at 4 weeks after placement. XPEED and SLA implant groups exhibited significantly higher BIC than the HA implant groups (*p* < 0.05). The XPEED implants had the highest BIC, followed by the SLA and HA implants. BA values (HA: 31.8 ± 6.8; SLA: 35.2 ± 5.9; and XPEED: 40.2 ± 6.5) showed no significant differences among the three implants (*p* > 0.05). Therefore, the null hypothesis was rejected because the Ti implants produced by the three different surfaces showed significant differences in BIC% values. The BIC% values showed differences in the results of this study, but the BA values revealed no significant differences between groups. The increased BIC% is related to osseointegration, in agreement with several studies reporting a direct correlation between BIC% and osseointegration; however, BA% showed no relation with osseointegration [31,55]. BIC% is defined as the length of the mineralized bone in direct contact with the threads. These findings supported the results of previous studies reporting significant osseointegration with Ca incorporation [30,31].

Therefore, these results indicated that the nanostructured, Ca-incorporated surface treatment (XPEED^®^) positively influenced implant stability, resulting in a larger BIC area and greater bone volume. The rough XPEED implant surface increased the surface area, thereby enlarging the BIC rate [56] and facilitating osteoblast growth, which plays a critical role in bone formation by adhering to the rough Ti surface in the early stages and gradually proliferating and differentiating, leading to osseointegration [57,58]. Nayab et al. [29] asserted that the bone healing mechanism associated with implant osseointegration involves the acceleration of extracellular matrix (ECM) proteins, which foster cell adhesion and the integrin-mediated osteoprecursor cell response on the implant surface. Moreover, as in the case of the XPEED implant with attached Ca ions, the implant surface nanostructure and the increased surface area with bivalent Ca concentration distributed over a 40% larger surface promote the adsorption of RGD-containing ECM proteins. Encouraging osteoblastic cell responses between the surface cell substrates and integrin-mediated osteoblastic cell responses on implant surfaces may support osteoblastic cell adhesion.

This study primarily focused on in vitro evaluations, limiting its ability to fully capture the complex interactions in the human body. Single-cell lines (commonly used in bone-related studies) may not fully represent the diverse cell types in the bone microenvironment. Furthermore, short-term evaluation may not provide a comprehensive understanding of long-term implant performance and stability. Evaluations in animal models, while informative, may not replicate the intricacies of human bone healing and immune responses. Additionally, while surface characterization techniques, such as XRD and XPS, provided valuable insights, more detailed techniques, such as AFM, could offer a deeper understanding of surface properties. Finally, while promising, the clinical relevance of the findings necessitates validation through well-designed human clinical trials considering various factors, such as patient demographics, surgical techniques, and postoperative care, to ensure applicability in real-world clinical settings.

## 4. Conclusions

The nanostructured, Ca-incorporated surface treatment (XPEED^®^) offers significant advantages in enhancing the performance of Ti implants, as demonstrated by the following key findings:

Enhanced early bone cell formation: The XPEED group exhibited a nanostructured Ca-coated surface, which may induce early bone cell formation by promoting protein interactions crucial for the initial stages of osseointegration.

Accelerated apatite formation: The XPEED surface demonstrated quicker apatite formation than the HA and SLA surfaces, indicating a more bioactive surface that could rapidly bond with natural bone.

Improved cell response and bioactivity: In vitro studies showed that the XPEED surface provided a more favorable environment for cell growth, proliferation, and viability, outperforming the HA and SLA surfaces in promoting osteoblastic activity.

Superior implant stability and osseointegration: The XPEED implants exhibited higher bone-to-implant contact (BIC) percentages and greater bone volume, indicating improved implant stability and better integration with the surrounding bone.

Higher removal torque values: The XPEED and SLA groups showed significantly higher removal torque values than the HA group, suggesting superior bone–implant integration and mechanical stability.

Homogeneous and dense mineralization: The XPEED surface demonstrated more homogeneous and densely mineralized bone deposition, highlighting its superior osteoconductive properties.

While these findings are promising, further research is necessary to fully understand the long-term effects and clinical implications of the XPEED surface treatment. Future studies should focus on long-term evaluations, detailed surface characterization techniques, and well-designed human clinical trials to validate the clinical relevance of our results and ensure their applicability in real-world settings.

## Figures and Tables

**Table 1:** Experimental groups of specimens in this study.

Group	Surface Treatment	Sample Shape
HA	Hydroxyapatite sandblasted	Disk shape (n = 25)
		Fixture (n = 16)
SLA	Alumina sandblasted + acid-etched	Disk shape (n = 25)
		Fixture (n = 16)
XPEED	Alumina sandblasted + acid-etched + calcium-coated	Disk shape (n = 25)
		Fixture (n = 16)

**Table 2:** Order, reagent, purity, and amount for preparation of m-SBF.

Order	Reagent	Purity (%)	Amount (g)
1	NaCl	>99.5	5.403
2	NaHCO_3_	>99.5	0.504
3	Na_2_CO_3_	>99.5	0.426
4	KCl	>99.5	0.225
5	K_2_HPO_4_·3H_2_O	>99.0	0.230
6	MgCl_2_·6H_2_O	>98.0	0.311
7	HEPES ^(a)^	>99.9	17.892
8	CaCl_2_	>95.0	0.293
9	Na_2_SO_4_	>99.0	0.072
10	1.0 M NaOH	-	≒15 mL

^(a)^ HEPES was previously dissolved in 100 mL of 0.2 M NaOH.

**Table 3:** The results for the surface roughness and contact angle values for the three Ti alloys (Mean ± SD, *n* = 6).

Group	Average Surface Roughness (μm)		Value of Contact Angle (°)
	Ra ^(a)^	Sa ^(b)^	
HA	1.92 ± 0.08	2.05 ± 0.14	123.7 ± 2.72
SLA	1.87 ± 0.11	1.92 ± 0.06	121.1 ± 2.20
XPEED	2.01 ± 0.13	2.12 ± 0.07	118.2 ± 1.91

^(a)^ Ra, average roughness of profile. ^(b)^ Sa, the center plane average. Note: There were no statistically significant differences in Ra, Sa, and contact angle values among all groups.

## Data Availability

The original contributions presented in the study are included in the article, further inquiries can be directed to the corresponding author.
